# The Inhibitors of CDK4/6 from a Library of Marine Compound Database: A Pharmacophore, ADMET, Molecular Docking and Molecular Dynamics Study

**DOI:** 10.3390/md20050319

**Published:** 2022-05-12

**Authors:** Lianxiang Luo, Qu Wang, Yinglin Liao

**Affiliations:** 1 The Marine Biomedical Research Institute, Guangdong Medical University, Zhanjiang 524023, China; 2 The Marine Biomedical Research Institute of Guangdong Zhanjiang, Zhanjiang 524023, China; 3 Southern Marine Science and Engineering Guangdong Laboratory (Zhanjiang), Zhanjiang 524023, China; 4 The First Clinical College, Guangdong Medical University, Zhanjiang 524023, China; wangqu0503@gdmu.edu.cn (Q.W.); lyl212608@gdmu.edu.cn (Y.L.)

**Keywords:** CDK4/6, marine compounds, pharmacophore construction, ADMET, molecular docking, molecular dynamics, mm-pbsa

## Abstract

Background: CDK4/6 (Cyclin-dependent kinases 4/6) are the key promoters of cell cycle transition from G1 phase to S phase. Thus, selective inhibition of CDK4/6 is a promising cancer treatment. Methods: A total of 52,765 marine natural products were screened for CDK4/6. To screen out better natural compounds, pharmacophore models were first generated, then the absorption, distribution, metabolism, elimination, and toxicity (ADMET) were tested, followed by molecular docking. Finally, molecular dynamics simulation was carried out to verify the binding characteristics of the selected compounds. Results: Eighty-seven marine small molecules were screened based on the pharmacophore model. Then, compounds **41369** and **50843** were selected according to the ADMET and molecular docking score for further kinetic simulation evaluation. Finally, through molecular dynamics analysis, it was confirmed that compound **50843** maintained a stable conformation with the target protein, so it has the opportunity to become an inhibitor of CDK4/6. Conclusion: Through structure-based pharmacophore modeling, ADMET, the molecular docking method and molecular dynamics (MD) simulation, marine natural compound **50843** was proposed as a promising marine inhibitor of CDK4/6.

## 1. Introduction

The uncontrolled cyclin D-CDK4/6-INK4-RB (Cyclin-dependent kinases 4/6-retinoblastoma protein) signaling pathway is usually associated with the abnormal proliferation of tumor cells [1]. Furthermore, in the G1 to S phases of the cell cycle, the uncontrolled signal pathway leads to the over phosphorylation of RB and the excessive separation of E2F transcription factors, which eventually leads to the uncontrollable proliferation of cells [2,3,4,5]. In addition, breast cancer is closely related to the anomalous expression of this pathway [6]. Therefore, inhibiting the expression of CDK4/6 can play a key role in the treatment of tumors [7,8]. Moreover, CDKs are a conserved kinase family [9]. Amino acid sequence analysis showed that CDK4 and CDK6 proteins have 71% similar sequences, which leads to their similar functions [10]. The crystal structures of CDK4/6 selected in this study and the Ramachandran plot are shown in Supplementary Figure S1. It is worth mentioning that the Ramachandran plot reflects the rationality of the selected CDK4 and CDK6 structures to a certain extent. The key amino acids at the active site of CDK4/6 ATP include His-95, Val-96, Asp-97 and Thr-102, and His-100, Val101, Asp-102 and Thr-107, respectively [11,12]. The confirmation of the above crystal structure and the key amino acids at the active site provides a structural basis for the discovery of CDK4/6 inhibitors.

At present, some achievements have been made in the development of CDK4/6 inhibitors, resulting in the emergence of many selective inhibitors: Palbociclib, Abemaciclib, Ribociclib, Trilaciclib and so on [13,14,15,16]. Clinical trials of these drugs in the treatment of cancer have also been carried out worldwide. Most clinical trials are the combination of CDK4/6 inhibitors and other drugs (such as tyrosine kinase inhibitors, HER2, EGFR inhibitors, PI3K kinase inhibitors, etc.) [17]. In addition, the latest generation of CDK inhibitors can selectively inhibit CDK4/6 while having little effect on other CDKs and maintain good anticancer effects while obtaining acceptable toxicity. However, drug resistance to approved CDK4/6 inhibitors has emerged and gradually increased [18].

Due to the vast marine environment, marine organisms are regarded as the most abundant source of bioactive natural products, and the compounds obtained from them reflect their biological diversity [19]. In the past few years, with the full exploitation of other resources, the marine environment has generated a new research field, and many drugs from marine natural products have entered clinical trials [20]. Recent studies have pointed out that 170 marine natural products and their synthetic analogues have strong anticancer biological activities [21]. In addition to anticancer activities, marine natural products have structural characteristics different from other environmental natural products and have a variety of biological activities, such as antibacterial, antiviral and antiinflammatory [22]. Therefore, marine natural products have received an increasing amount of attention from scientists. Natural products are the best choice for the source of new drugs [23,24]. We collected three marine natural product databases. The Seaweed Metabolite Database (SWMD), Comprehensive Marine Natural Product Database (CMNPD) and Marine Natural Product Database (MNPD) [25,26,27], and screened two small molecules of marine origin with CDK4/6 inhibitory potential from the databases by a series of computer-assisted methods.

**Figure 1:**
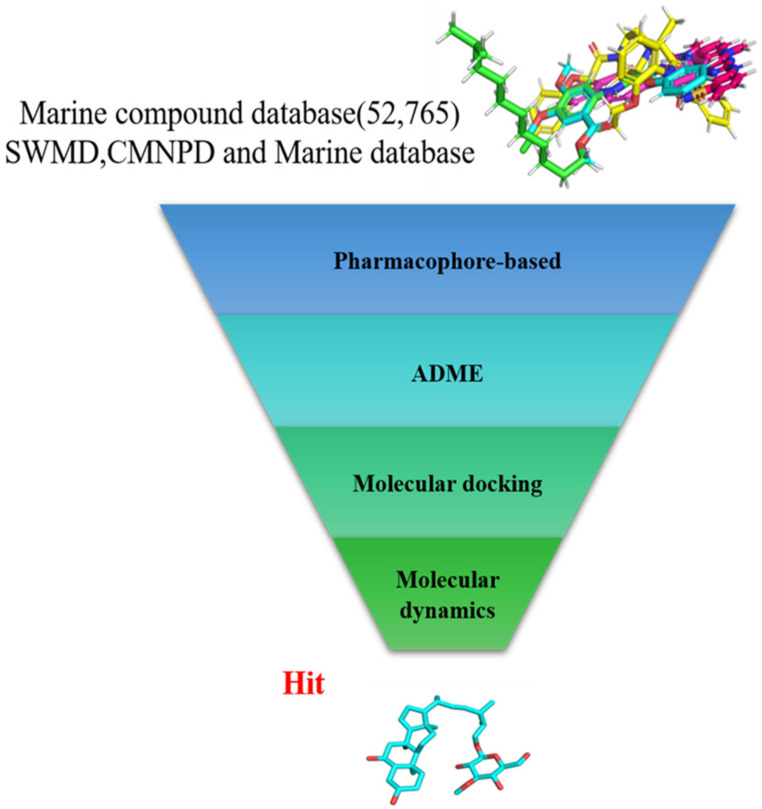
Workflow of this study: marine compound database construction, a pharmacophore, ADMET, molecular docking, and molecular dynamics.

In the present study, we found two new chemical inhibitors with CDK4/6 inhibitory properties by pharmacophore modeling, ADMET property prediction, molecular docking and molecular dynamics (MD) simulation methods [28] (Figure 1).

Considering that CDK4 is a dimer and that the active residues of both proteins are mainly in the A-chain, we only compared the stacked A-chain structures of CDK4 and CDK6. The Alignment C-α distance cutoff was set as 2.5, and length cutoff was set as 50. The RMSD (in angstrom) for the two protein structures was 1.1380. It is generally accepted that a smaller RMSD value means a higher overlap in protein spatial structure, and an RMSD value of less than 2 Å is good [29].

**Figure 2:**
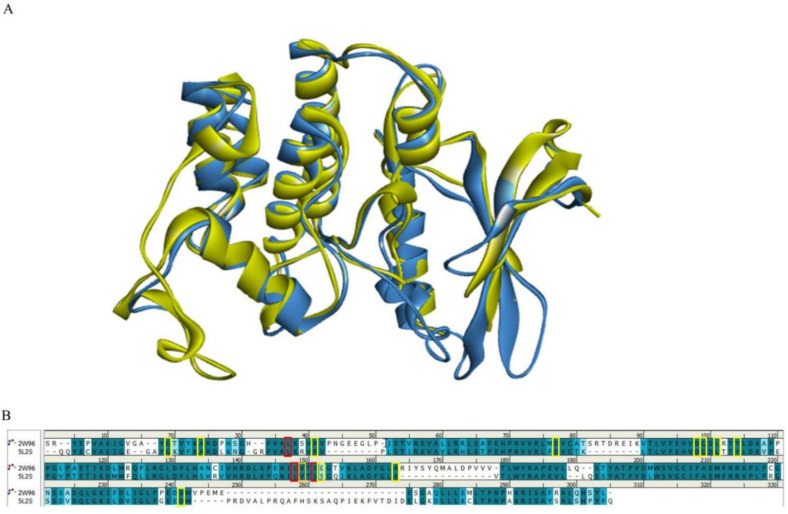
Comparison of CDK4/6 protein structures and key residues. (**A**) Schematic representation of the superimposed CDK4 and CDK6 structures. The CDK4 (PDB: 2W96) structure is shown in blue and the CDK6 (PDB: 5L2S) structure is shown in yellow. (**B**) Comparison of the amino acid sequences of CDK4 (PDB: 2W96) and CDK6 (PDB: 5L2S). Residues defining the active site boundary are highlighted in red for the PDB structure of CDK4 and in yellow for the PDB structure of CDK6.

Figure 2 shows a comparison of the A-chain sequences of CDK4 and CDK6. The PDB structure-defining active site boundary residues of CDK4 are highlighted in red and the PDB structure-defining active site boundary residues of CDK6 are highlighted in yellow. The darker cyan residue pairs represent that they are identical, the lighter the blue the greater the difference between the residue pairs and the white residue pairs represent that they are completely different in origin. It is easy to see that the active site boundary residues of both structural A-chains are almost always highly homologous. It is worth noting that we are studying here the protein structure active residues published by PDB.

**Figure 3:**
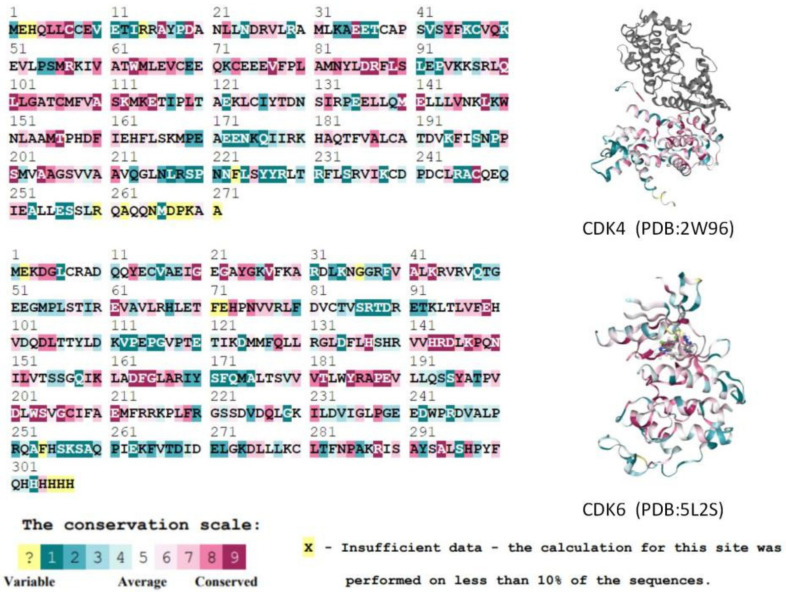
Comparison of CDK4 and CDK6 protein A-chain residue sequences and conservativeness. Higher scoring levels indicate higher conservativeness of residues. In this case, the yellow residues (which do not contain the active residues of the proteins) could not be classified by the conservativeness grade due to their low frequency of occurrence in the database.

In addition, we calculated evolutionary conserved scores for residues using the ConSurf-DB online tool for two selected CDK protein structures (CDK4: 2W96; CDK6: 5L2S). Consecutive conserved scores were divided into discrete classes of nine levels, with level 1 indicating the most variable residues and level 9 indicating the most conserved position. Figure 3 shows the conservativeness of the sequences of residues in the A-chain of the CDK4 and CDK6 proteins. Key residues in the ATP-binding pocket of the CDK4/6 proteins [11,12] are all considered to have a moderate (class 5) or higher conservativeness score. In summary, it is known that the active binding site residues of CDK4/6 are highly homologous and conserved, and thus do not affect the binding of any ligands to the protein.

## 2. Results

### 2.1. Pharmacophore Models: Construction, Selection and Application

**Figure 4:**
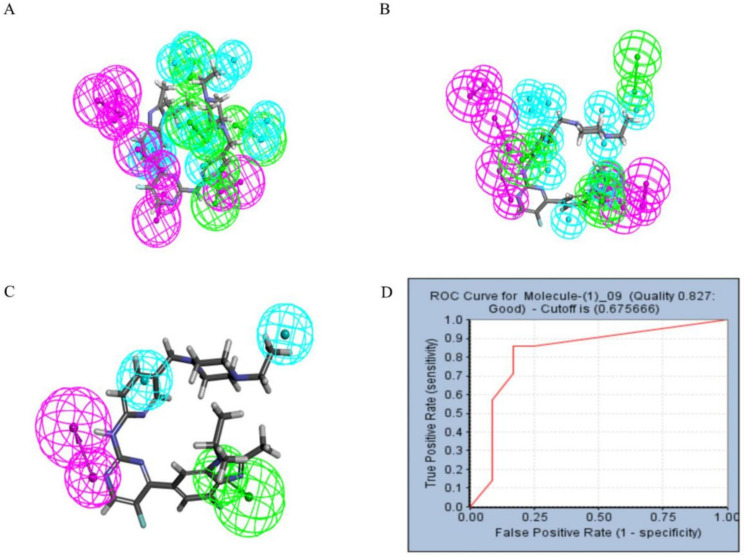
The pharmacophore model and receiver operating characteristic (ROC) curve validation. Hydrophobic group features are shown as blue spheres, hydrogen bond acceptor features are shown as purple spheres and hydrogen bond donor features are shown as green spheres. (**A**) Pharmacophore model 01–05. (**B**) Pharmacophore model 06–10. (**C**) Coincidence effect drawing of Abemaciclib and pharmacophore 09. (**D**) ROC curve.

Pharmacophore models can be used to simulate the active conformation of ligand molecules through conformational search and molecular superimposition and can be used to infer and interpret possible interaction patterns between receptor and ligand molecules. Ten common feature pharmacophore models were built with the Discovery Studio platform. The results show that all 10 pharmacophore models have good active/inactive molecule recognition. Figure 4a shows the models 01–05 and Figure 4b illustrates the pharmacophore models 06–10. As shown in Table 1, the pharmacophore model Phar09 (HHDA) had the lowest false positive and false negative error rates and the highest sensitivity for the test set molecules. In addition, the area under the curve (AUC) for Phar09 was 0.827, confirming the model’s ability to identify positive molecules well (Figure 4d). The superimposed effect of Abemaciclib with Phar09 can be seen in Figure 4c. A library of 50,000 molecules of marine compounds was screened with the model Phar09. All molecules were pretreated with the Prepare Ligand program. The marine drug library was screened using Pharmacophore 09, and 87 molecules with a fit value greater than 3.50 were selected for further study.

### 2.2. Absorption, Distribution, Metabolism, Excretion, and Toxicity (ADMET) Analysis

The pharmacokinetic method of ADMET (drug absorption, distribution, metabolism, excretion, and toxicity) is important in drug design and drug screening. We built a prediction process in Discovery Studio. We analyzed the ADMET descriptors of 87 marine molecules selected by pharmacophore. This research was carried out through the Calculate Molecular Properties function of the Discovery Studio platform. The blood–brain barrier permeability (BBB), water solubility, intestinal absorbance, hepatotoxicity and CYP2D6 enzyme inhibition descriptors of the drug were predicted. The blood–brain barrier permeability of all compounds was predicted to be “undefined”, indicating that the blood–brain barrier permeability of all 20 compounds was outside of the 99% confidence ellipse [30]. Except for the blood–brain barrier permeability of the drug not being successfully predicted, the rest of the properties were described in a numerical or hierarchical manner. To efficiently select the molecules with better medicinal properties, we removed all the compounds with hepatotoxicity and CYP2D6 inhibition, and the water solubility, intestinal absorbance, hepatotoxicity and CYP2D6 inhibition values of the remaining 20 molecules are shown in Table 2. Among them, the range of water solubility grade is 1–5. The higher the grade goes, the better solubility the water has. The intestinal absorption rate is also divided into 1–5 grades. For hepatotoxicity and CYP2D6 enzyme inhibition, the negative value of the compound is inversely proportional to the cytochrome enzyme inhibition activity. Twenty compounds were predicted to have moderate water solubility and intestinal absorption; their hepatotoxicity and cytochrome enzyme inhibition was low, suggesting that they have good proprietary medicine properties.

### 2.3. Molecular Docking

Molecular docking can be used to explore the optimal binding mode between compounds and targets. Therefore, to further screen the compounds with good target inhibitory activity, we used CDK4 and CDK6 protein structures as targets for Libdock [31] molecular docking, and their Libdock scores and pharmacophore-screened Fit Values are listed in Table 3. The active sites of CDK4 (PDB ID:2W96) were Asp-99, Asp-140, Lys-142, Tyr-17 and Thr-172. The docking radius was set to 17. CDK6 (PDB ID:5L2S) took the original ligand as the center and set the sphere with a docking radius of 10 as the active site. The structure of the protein was optimized and hydrogenated by the Clean Protein program in advance. Docking preference was set as high quality, the number of spatial hotspots was set to 100 and the ligand conformation generation method was BEST to perform operations. To distinguish and determine which molecules had the better target binding activity, we selected the positive molecule Abemaciclib for the molecular docking study, and compounds with docking scores higher than Abemaciclib were considered valuable for further studies. The results, as shown in Table 3, indicate that all 20 molecules and CDK6 structures had higher Libdock scores, and seven of them had better scores than positive controls; but, generally speaking, there was no significant difference in docking scores between these 20 molecules and Abemaciclib. However, only three molecules bound to the active residues of the CDK4 structure, which were molecules 20551, **50843** and **41369**.The docking scores of the latter two were higher than those of the positive control Abemaciclib, and they also had better docking scores with the CDK6 structure. Therefore, we chose the two molecules to dock with the two targets with higher precision CDOCKER and analyzed the interaction force between them in detail.

**Figure 5:**
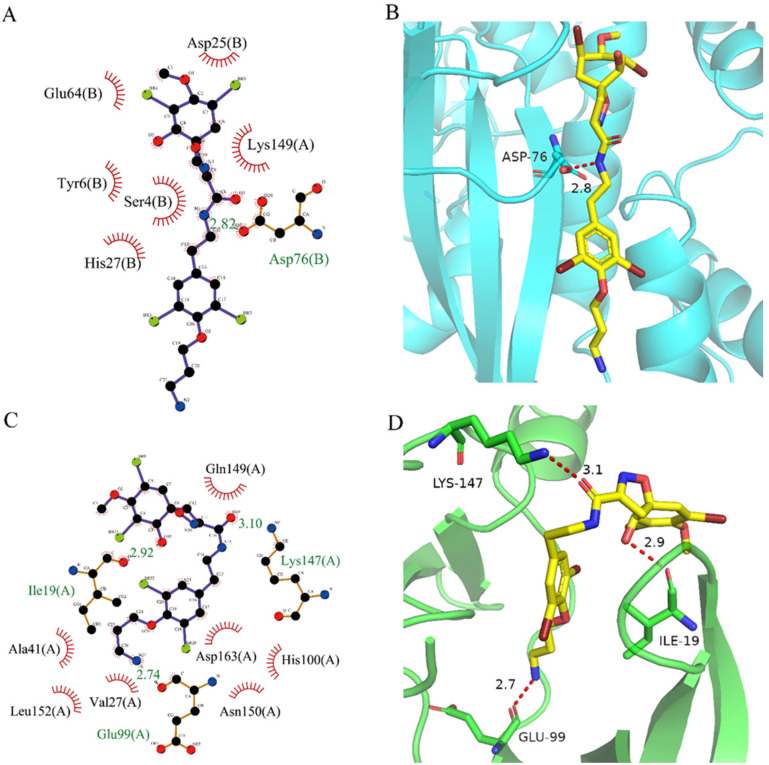
Analysis of binding mode between compound **41369** and CDK4/6. (**A**) A 2D interaction schematic of compound **41369** with CDK4. Hydrogen bonds are shown as green dashed lines, hydrophobicity is in red lines. (**B**) A 3D binding mode of compound **41369** with CDK4. Hydrogen bonds are shown as red dashed lines, while compound **41369** is shown in golden yellow. (**C**) A 2D interaction schematic of compound **41369** with CDK6. Hydrogen bonds are shown as green dashed lines, hydrophobicity is in red lines. (**D**) A 3D binding mode of compound **41369** with CDK6. Hydrogen bonds are shown as red dashed lines, while compound **50843** is shown in golden yellow.

**Figure 6:**
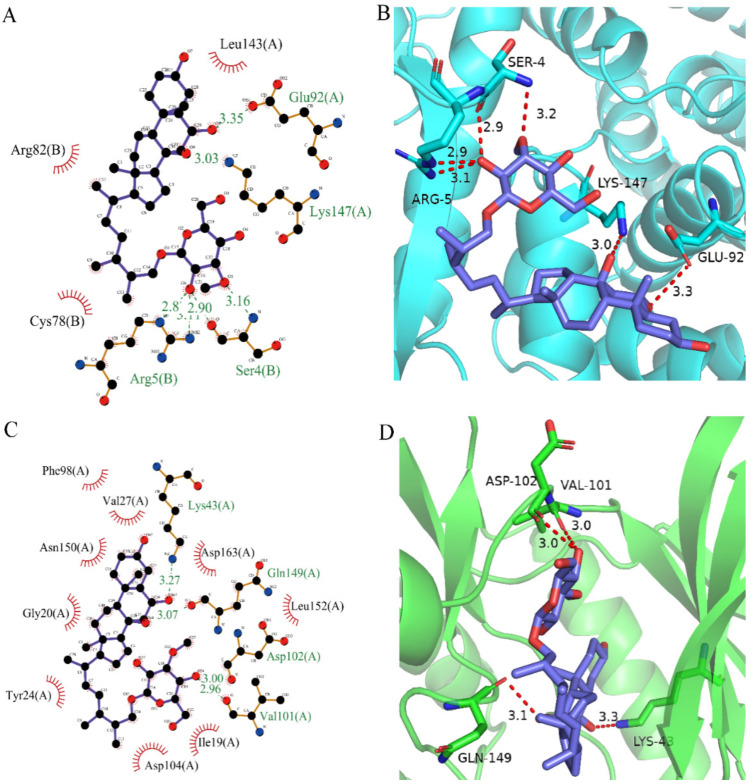
Analysis of binding mode between compound **50843** and CDK4/6. (**A**) A 2D interaction schematic of compound **50843** with CDK4. Hydrogen bonds are shown as green dashed lines, hydrophobicity is in red lines. (**B**) A 3D binding mode of compound **50843** with CDK4. Hydrogen bonds are shown as red dashed lines, while compound **50843** is shown in blue. (**C**) A 2D interaction schematic of compound **50843** with CDK6. Hydrogen bonds are shown as green dashed lines, hydrophobicity is in red lines. (**D**) A 3D binding mode of compound **50843** with CDK6. Hydrogen bonds are shown as red dashed lines, while compound **50843** is shown in blue.

After analyzing and comparing the docking scores (Table 3), we selected the first two molecules with the highest sum of docking scores with the two targets and used the CDOCKER program to study their interaction with the receptors. The two molecules were finely docked. In the CDOCKER docking program, we used the charmM force field to deal with the protein structure and ligands. Finally, the docking results of compounds **41369** and **50843** with CDK4/6 are shown in Figure 5 and Figure 6. The interaction diagram (Figure 5a) and three-dimensional binding pattern diagram (Figure 5b) of compound **41369** with CDK4 shows that the compound formed hydrogen bonding interactions with the side chain of protein B-chain residue Asp-76. For CDK6, compound **41369** formed hydrogen bonding interactions with the side chain of residue Lys-147 of the A-chain, the backbone of residues Ile-19 and Glu-99 (Figure 5c). Furthermore, the three-dimensional docking pattern of CDK6 with compound **41369** is shown in Figure 5d. The hydrogen bonding interaction between N and O on more residues indicates that compound **41369** and CDK4/6 had some interaction force. Furthermore, as seen in Figure 6a, compound **50843** formed hydrogen bonding interactions with residues Lys-147, Glu-92, Ser-4 and Arg-5 of CDK4, indicating tight binding (Figure 6b). For CDK6, compound **50843** formed hydrogen bonds with the side chain of A-chain residue Lys-43, and the backbone of residues Gln-149, Asp-102 and Val-101 (Figure 6c). Similarly, the three-dimensional docking pattern of CDK6 with compound **50843** is also shown in Figure 6d. Combined with the reported results, the A-chain residue Val-101 is necessary for the binding of CDK6 to the inhibitor, which could also provide guidance for future chemical optimization of this compound.

### 2.4. RMSD and RMSF Analysis

**Figure 7:**
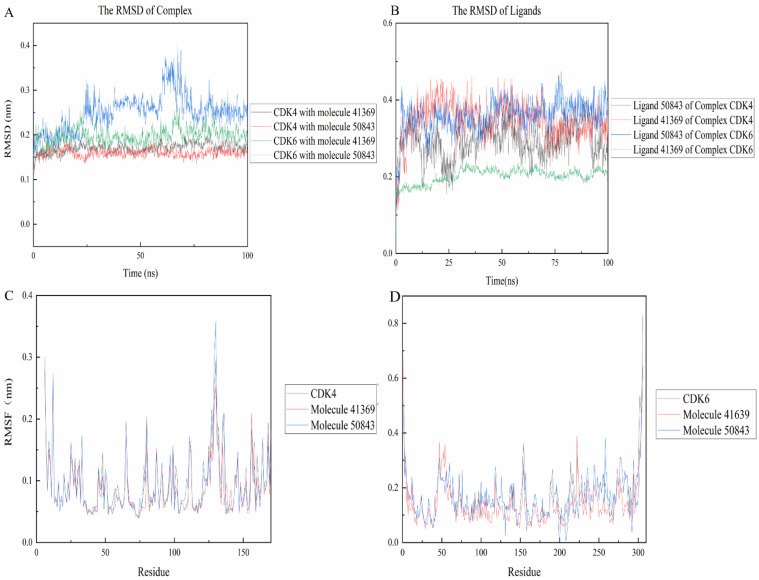
RMSD and RMSF plots of compound **50843** and **41369** with CDK4/6. (**A**) The RMSD of complexes. CDK4 and compound **41369** is shown as black lines, CDK4 and compound **50843** is in the red line, CDK6 and compound **41369** is shown as blue lines, CDK6 and compound **50843** is in the green line. (**B**) The RMSD of ligands. Ligand **50843** of complex CDK4 is shown as black lines, Ligand **41369** of complex CDK4 is shown as red lines, Ligand **50843** of complex CDK6 is shown as blue lines, Ligand **41369** of complex CDK6 is shown as green lines. (**C**) The RMSF of complexes and CDK4. CDK4 is shown as black lines, compound **41369** is in the red line and compound **50843** is in the blue line. (**D**) The RMSF of complexes and CDK6. CDK6 is shown as black lines, compound **41369** is in the red line and compound **50843** is in the blue line.

The RMSD between two protein structures was used to describe the differences in their atomic positions, which can reflect the stability of the whole system. As shown in Figure 7, the complexes were more stable than their own receptor in the 100 ns simulation, while CDK4 and compound **50843** ended up stable at 0.15 nm and the simulation process did not fluctuate too much, and the same compound **41369** finally stabilized at 0.17 nm (Figure 7a). For CDK6, we found that the **41369** complex fluctuated at the beginning of 57 ns and finally stabilized at 62 ns with the RMSD value being 0.25 nm. Interestingly, the RMSD value of the **50843** complex did not fluctuate too much in the process of the 100 ns simulation and the RMSD value was 0.2 nm. The last four systems were able to reach a stable state in the simulation process. For the RMSD values of ligands in the system, compounds **41369** and **50843** remained stable in the simulation process of 100 ns and finally balanced in an appropriate range (Figure 7b).

The RMSF refers to the root mean square displacement of each amino acid of a certain frame conformation compared with the average conformation, which is used to determine the flexibility of a region of a protein. Firstly, it can be seen from Figure 7c that the three systems all show high RMSF values near the binding pocket, which to a certain extent indicates that the pocket is more flexible. In contrast, compound **50843** increases the flexibility of the pocket slightly, but the RMSF value of the pocket is within an acceptable range; for CDK6 we focus on residue Val-101, compound **50843** has a hydrogen bond with Val-101 of CDK6. It was found that the RMSF value of val-101 decreases (Figure 7d). In terms of flexibility, the four complex systems exhibited minor fluctuation, and the comprehensive RMSD and RMSF compounds **41369** and **50843** could stably bind to CDK4/6.

### 2.5. The Hydrogen Bond Analysis

**Figure 8:**
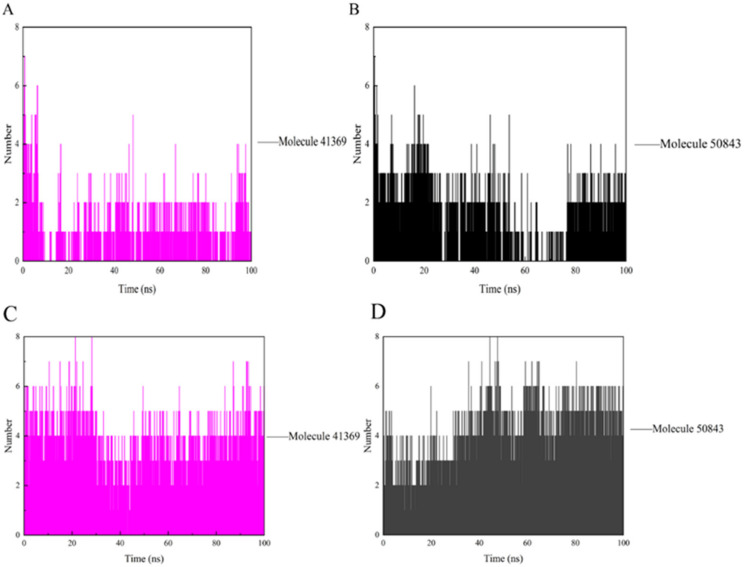
The hydrogen bond of CDK4/6 with compound **41369** and **50843**. (**A**) Compound **41369** (magenta) with CDK4. (**B**) Compound **50843** (black) with CDK4. (**C**) Compound **41369** (magenta) with CDK6. (**D**) Compound **50843** (black) with CDK6.

During the simulation, the ligand formed a certain number of hydrogen bonds with the protein, and the number and survival time of these hydrogen bonds also reflected the binding degree of the ligand to the protein. In Figure 8a, compound **41369** had too few hydrogen bonds for a period of time in the simulation, while compound **50843** formed more hydrogen bonds with CDK4 overall and survived longer (Figure 8b). As for CDK6, there was always hydrogen bond interaction during the complex system in the 100 ns simulation process. The hydrogen bond formed by compound **41369** and CDK6 fluctuated at the simulated 40 ns (Figure 8c). In Figure 8d, it is shown that CDK6 and compound **50843** fluctuated in the early stages of the simulation, and the hydrogen bond fluctuated in the simulation process. It is able to be seen from these figures that compounds **41369** and **50843** formed hydrogen bonds with the CDK4/6 simulation process and survived for a long time.

### 2.6. Solvent Accessible Surface Area and Radius of Gyration Analysis

**Figure 9:**
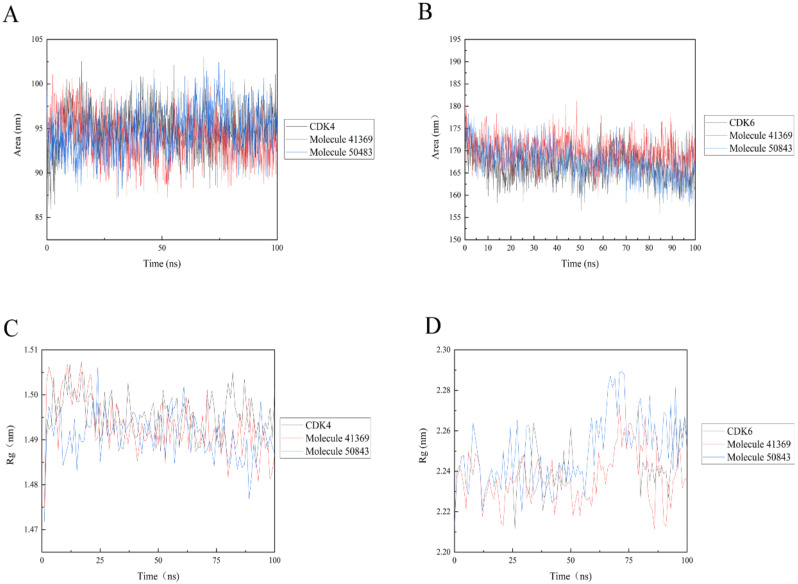
Solvent accessible surface area (SASA) and radius of gyration (Rg) of 100 ns simulation process. (**A**) SASA plots of compound **50843** (blue) and **41369** (red) with CDK4 (black). (**B**) SASA plots of compound **50843** (blue) and **41369** (red) with CDK6 (black). (**C**) Rg plots of compound **50843** (blue) and **41369** (red) with CDK4 (black). (**D**) Rg plots of compound **50843** (blue) and **41369** (red) with CDK6 (black).

Solvent accessible surface area (SASA) analysis can be used to indicate the solvent accessible surface area of the entire complex. The final simulation results show that the SASA values of the protein complexes (see Figure 9) are relatively stable during the trajectory; in Figure 9a, the complex systems all show lower SASA values. The complex system of CDK6 shows a higher SASA value, indicating that the complex system of compounds **41369** and **50843** with CDK6 found it easier to approach the solvent (Figure 9b).

The radius of gyration (Rg) indicates the firmness of the protein structure. As shown in Figure 9, the average Rg values of CDK4 with compounds **50843** and **41369** were lower than 2.3 nm (Figure 9c), while a similar pattern was shown in the systems complexed with CDK6, and the average Rg values of these systems were lower than 1.5 nanometer; it is worth mentioning that the Rg value of compound **50843** with the CDK6 system was lower (Figure 9d). The results show that the compactness of the complex was maintained during the simulation.

### 2.7. Protein–Ligand Interaction Energy Analysis

**Figure 10:**
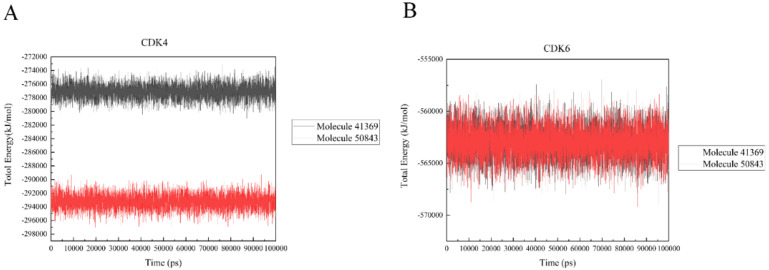
The total energy of CDK4/6 with molecule **41369** and **50843**. (**A**) The total energy of compound **41369** (black) and **50843** (red) with CDK4. (**B**) The total energy of compound **41369** (black) and **50843** (red) with CDK6.

In a bid to quantify the interaction strength between the ligand and receptor, the non-bonding interaction energy between the two needs to be calculated; we used the gmx_energy program to calculate the energy change of the ligand and protein during the simulation. It is vital to note that the values here are not free energies or binding energies. Figure 10a shows that the estimated total energy value between compound **41369** and CDK4 is −277,000 kJ/mol, while the total energy value of compound **50843** is −293,000 kJ/mol; CDK6 and compounds **41369** and **50843** are both −562,000 kJ/mol (Figure 10b). The results show that the interaction of compound **50843** with CDK4/6 is stronger in this energy numerical calculation.

### 2.8. MM-PBSA Analysis

**Figure 11:**
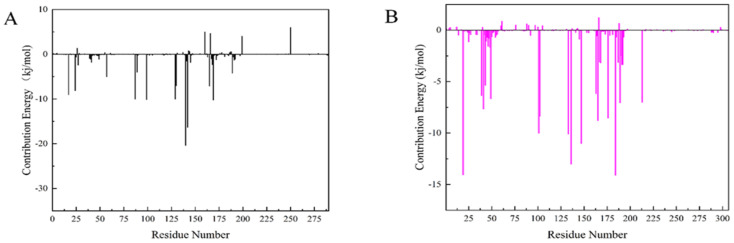
Residue decomposition diagram of binding energy. (**A**) Compound **50843** with CDK4 (black). (**B**) Compound **50843** with CDK6 (magenta).

The Molecular mechanics Poisson Boltzmann surface area (MM-PBSA) method is prevalent in the estimation of the free binding energy between CDK4 and CDK6 with compound **50843**. From the aspects of structural stability and flexibility and the simulation results of quantitative interaction, we finally selected compound **50843** for MM-PBSA calculation. The binding free energy component of each complex with compound **50843** was calculated in a time step of 1 ns in a molecular dynamics simulation of 100 ns. It can be seen from Table 4 that the binding free energies of compound **50843** and CDK4/6 are −154.655 kJ/mol and −212.082 kJ/mol respectively, which are also contributed to by van der Waals force to a great extent. Therefore, according to the energy calculation of MM-PBSA, a stronger binding interaction with protein was established. To further analyze the interaction between compound **50843** and protein, we decomposed the binding free energy of MM-PBSA into the energy contribution of each protein residue to evaluate the key binding residues; that is, the residues with higher energy contribution to the binding free energy. It can be seen from Figure 11a that the key residues in compound **50843** and CDK4 are Arg-5, Ser-4, Met-17, Arg-87, Asp-129, Asn-130, Leu-142 and Pro-169. Interestingly, the residues forming the hydrogen bond interaction in molecular docking appear in it. For Cdk6 and compound **50843**, their key residues include Ile-19, Ala-41, Val-101, Leu-133, Leu-136, Lys-147, Asp-163, Gly-165 and Glu-189. It is worth mentioning that the binding energy contributed by residue val-101 is −10.0042 kJ/mol (Figure 11b). The results show that MM-PBSA not only verified the results of molecular docking but also further quantified the binding energy between compound **50843** and CDK4/6.

### 2.9. Analysis of Synthetic Accessibility Score Parameters

To explore the further synthetic and application potential of these two compounds, the SA scores of the compounds calculated using ADMETlab were used to assess the synthesizability of the two alternative compounds. As shown in Table 5, the SA scores for compound **41369** and compound **50843** were 5.226 and 5.517, respectively. Lower SA scores tend to imply better synthesizability. Although not as good as the positive control, the two compounds we selected still had good SA scores, suggesting their potential to be synthesized as new drugs.

### 2.10. Prediction of Inhibitory Activity of Tumor Cell Lines

To predict the potential inhibitory activity of two candidate compounds against tumor cell lines, the way2durg online tool was applied. Both Pi and Pa of the two compounds were predicted. The results showed that both compounds exhibited inhibitory activity against MDA-MB-231 and HL-60 cell lines, respectively (Table 6).

## 3. Discussion

In recent years, CDK4/6 inhibitors have continued to be applied to the clinical trials of various malignant tumors. However, these inhibitors have some disadvantages, such as gastrointestinal toxicity and drug resistance, which lead to the decline in their efficacy. At present, drug research and development are also taking more heed of the existing natural products in nature, and marine natural products have been of interest to the scientific community for their unique ecological advantages. Therefore, our study aims to screen CDK4/6 compounds using marine natural products.

In this study, we collected some listed CDK4/6 inhibitors by reading the literature, constructed a structure-based pharmacophore model using Discovery Studio and selected model 09 with the highest selection score; in addition, we verified the model. AUC under the ROC curve reflects the discrimination ability of the model. It is worth mentioning that some studies also use the QSAR model to screen a marine natural product, and our pharmacophore modeling was based on the selected known inhibitors, instead of structured modeling on the complex. The marine natural product compounds obtained from the pharmacophore model generated by this method can be more appropriate to the known CDK4/6 inhibitors in terms of conformation [32,33]. Then we carried out the ADMET test, and finally obtained 20 compounds that showed good ADMET characteristics.

In the molecular docking study, compound Abemaciclib was selected as the positive control, and then docking was carried out by the Libdock program. Finally, compounds **41369** and **50843** were selected according to the comprehensive scoring performance of compounds and CDK4/6, and their binding modes were further analyzed by a more precise CDOCKER program. It was found that compound **50843** could form a hydrogen bond with val-101 of CDK6, this residue has an important relationship with CDK6, and compound **41369** also formed a close interaction with CDK4/6 [34].

Molecular dynamics is of great significance to confirm the stability of binding between compounds and proteins. After the 100 ns simulation, we collected the RMSD and RMSF data of the trajectory to confirm the stability and flexibility of the binding between the compound and protein. In addition, we also analyzed the stability of the whole complex system in terms of the hydrogen bond, SASA and Rg. Distinct from some previous studies, we analyzed the stability from many aspects. For the interaction and free binding energy between compounds and proteins, we first used the total energy of the system to quantify the interaction between compounds **41369** and **50843** and CDK4/6. Interestingly, compound **50843** performed much better in the comparison of interaction strength [35]. Finally, we selected the trajectories of compound **50843** and CDK4/6 for MM-PBSA calculation. In the residue energy contribution, we found some key residues not mentioned in previous studies, such as Arg-5, Ser-4 and Pro-169 of CDK4 and Asp-163, Gly-165 and Glu-189 of CDK6. Of course, Val-101 of CDK6 contributes a very high negative binding energy, further indicating the important relationship of compound **50843** for CDK6.

Finally, we carried out some studies on the synthesis and cytotoxicity of compounds **50843** and **41369**. In terms of results, SA score shows that compounds **50843** and **41369** can be synthesized, and their toxicity is also within an acceptable range, which also provides some reference data for subsequent experimental verification.

Briefly, marine natural products have made an immense contribution to the pharmacy domain owing to its ample resources. Through the method of computer-aided drug design, CDK4/6 inhibitors were quickly found from 52,765 marine natural products. In addition, compound **50843** can be optimized as a crucial lead compound to become a better CDK4/6 inhibitor.

## 4. Materials and Methods

### 4.1. Database Construction and Molecular Preparation

Seaweed Metabolite Database (http://www.swmd.co.in, accessed on 1 April 2021), Comprehensive Marine Natural Products Database (https://www.cmnpd.org, accessed on 1 April 2021) and Marine Database (http://docking.umh.es/, accessed on 1 April 2021) were integrated into one database. The database, which included 50,000 marine compounds, was saved in mol format in advance and imported into the Discovery Studio platform. The internal database of the platform was constructed with the help of the function of constructing a 3D database. Compounds with erroneous valence states were removed and 3D coordinates were generated for the rest of the molecules. The resulting database contained all the molecules of the three databases. Finally, the structure of the compound in the database was repaired by the Prepare Ligand tool in Discovery Studio. ConSurf-DB online tool [36] was used to calculate the evolutionary conserved scores of CDK4/6 structures.

### 4.2. Pharmacophore Construction

Using the Discovery Studio platform, five CDK4/6 inhibitors that have entered clinical studies [13,14,15,16] **(**Supplementary Figure S2) were superimposed and 10 pharmacophore hypotheses were generated based on their molecular common features. For each generated hypothesis, we set the features it could contain to be hydrogen bond acceptors(A), hydrogen bond donors(D) and hydrophobic features(H), and each pharmacophore hypothesis contained up to five of the same feature. The minimum distance between pharmacophore features within the model was set at 2.97 Å and the best conformation method was applied to generate potential conformations for the positive compounds.

In addition, seven newly published inhibitors from Li et al. were selected as active molecules for testing [37], and 12 inactive decoy molecules were generated using the DUD-E online tool for all inhibitors (five marketed and seven newly published structures, shown in Supplementary Figure S2 and Supplementary Table S1). The DUD-E online decoy generation tool preserved the backbone structure of the positive inhibitors better, allowing the generated inactive molecules to maintain maximum structural similarity to the positive inhibitor [38]. The discriminatory ability and sensitivity of the pharmacophore model was characterized by subject operating characteristic (ROC) curves. The X-axis of the horizontal coordinate of the curve plot is 1-specificity, i.e., the false positive rate. The closer the X-axis is to zero, the higher the accuracy; the Y-axis of the vertical coordinate is called sensitivity, also known as the true positive rate (sensitivity), and the larger the Y-axis is, the higher the accuracy, as reflected by the area under the ROC curve (AUC) being closer to 1 [39]. The pharmacophore ranking score given by the platform was also considered, and the pharmacophore with both the optimal ranking score and the area under the ROC curve (AUC) value was selected as the next step in the virtual screening [30]. The structures of all inhibitor and decoy molecules are shown in Figure S2 and Table S1 of the Supplementary Material.

### 4.3. Absorption, Distribution, Metabolism, Elimination, and Toxicity (ADMET)

Pharmacokinetics and toxicity (ADMET) assessment is helpful to screen and eliminate molecules with poor proprietary properties from a large number of compounds. Using Discovery Studio’s Calculate Molecule Properties tool, we calculated the ADME properties of 87 compounds. Four pharmacokinetic parameters, including intestinal absorption (HIA), hepatotoxicity (hepatoxic), cytochrome CYP2D6 inhibitory activity and water solubility at 25 °C, were predicted. CYP2D6 plays an important role in the degradation of drugs in vivo. Better intestinal absorbance and CYP2D6 inhibitory activity can prolong the action time of drugs in the human body to the greatest extent. We removed the compounds with poor water solubility, intestinal absorbance and CYP2D6 inhibition, and selected the remaining compounds for further study.

### 4.4. Molecular Docking

The molecular docking program consists of two parts. First of all, to quickly select the molecules with good target binding activity, 20 molecules selected by ADMET were docked with CDK4 and CDK6, respectively. Among them, the binding site of CDK4 (PDB:5L2S) was determined by the original ligand, the residue of the binding site of CDK6 (PDB:2W96) was identified as Asp-99, Asp-140, Lys-142 and Tyr-17, the central coordinate of the docking site was 24.870297, 18.922378 and 10.713189, and the radius of the two receptor structures was determined to be 15. Then, we used the CDOCKER program to fine dock two molecules with good Libdock scores with the two targets and analyze the interaction between them and the target. Pose Cluster Radius was set to 0.1, and the number of conformations randomly generated by ligands was 10, to achieve the purpose of ligand–receptor semi-flexible docking.

### 4.5. Molecular Dynamics 

After docking, the 100 ns molecular dynamics simulation was used to simulate the complex model of CDK4 and CDK6 with compound **50843** and compound **41369** to test the stability and flexibility of the complex. In this study, the GROMACS2018.1 software package, AMBER99SB-ILDN force field, and SPC216 water model were used for molecular dynamics simulation [40,41]. To ensure the total charge neutrality of the simulation system, a corresponding number of sodium ions were added to the system to replace water molecules to form a solvent box of appropriate size. Then the periodic boundary condition (PBC) was applied to three directions of the system. Using the AMBER99SB-ILDN force field, the force field parameters of the compound were obtained from the ACPYPE website (https://www.bio2byte.be/acpype/, accessed on 3 May 2021) [42]. Initially, the energy of 50,000 steps of the whole system was minimized (EM) at 300 K. After energy minimization, in order to maintain the pressure and temperature of the system, the two minimized systems were balanced by position constrained MD simulation at 300 K for 100 ps. After balancing, the four systems all carried out the final production operation of 100 ns MD simulation at 300 K. The Berendsen coupling algorithm was used to apply periodic boundary conditions under isothermal and isobaric conditions, and the pressure was set to 1 atm. In these four systems, Lin CS algorithm was used to constrain hydrogen bonds. The electrostatic interaction was analyzed by particle grid Ewald method. The calculation of van der Waals Coulomb interaction took 1.3 nm as the cutoff point. The whole MD track was recorded every 100 picoseconds (PS) [43,44,45]. Finally, the root means square deviation (RMSD) of the system and the root mean square fluctuation of atomic position (RMSF) were analyzed. In addition, the radius of gyration (Rg), the solvent accessible surface area (SASA), the total potential energy change curve and the number of hydrogen bonds of each system were also collected [46].

### 4.6. MM-PBSA

MM/PBSA method is widely used in the calculation of free energy of receptor–ligand binding. This method is called Molecular Mechanics/Poisson Boltzmann (Generalized Born) Surface Area [47]. The basic principle is to calculate the difference between the bound and unbound free energies of two solvated molecules or to compare the free energies of different solvated conformations of the same molecule. We extracted a stable 10 ns from the trajectory for calculation. The following Equation (1) below describes the binding free energy, and the resulting output formula is related to the calculated energies of the ligand and receptor.

(1) $$G_{binding}=G_{protein}-\left(G_{complex}+G_{ligand}\right)$$

The free energy of the protein–ligand complex is expressed by *G_Complex_*, *G_protein_* represents the free energy of the protein in the solvent and *G_ligand_* represents the free energy of the ligand in the solvent.

### 4.7. Analysis of Synthetic Accessibility Score Parameters

The SA score was calculated for the two best compounds using the ADMETlab 2.0 (public by Guoli Xiong et al., Xiangya School of Pharmaceutical Sciences, Central South University, Changsha Hunan, China) online platform [48], considering that the two compounds we selected were not existing available molecules. The SA Score is based on the “complexity” of the molecule, weighting the frequencies of the ECFP4 fingerprints of 1 million compounds obtained from PubChem and summing them to obtain a fragment Score, assuming that “frequently occurring substructures are easy to synthesize”. Meanwhile, the “Complexity Penalty” takes into account the molecular weight and complexity of the compound and normalizes the value from 1 (easy) to 10 (difficult), as is shown in Equation (2). A lower SA score implying that the compound is easier to synthesize.

(2) SA score=fragment score−complexity Penalty

### 4.8. Prediction of Inhibitory Activity of Tumor Cell Lines

We used the way2durg online tool to predict the inhibitory activity of two alternative compounds against tumor cell lines. This analysis was performed by using the CLC-Pred web facility [48]. The activity assessment of both compounds was performed based on QSAR models built on the Prediction of Substance Activity Spectra (PASS) tool (http://www.way2drug.com/PASSonline, accessed on 14 April 2022) and a training dataset based on ChEMBLdb cytotoxicity data.

## 5. Conclusions

In conclusion, in this study, compound **50843** was screened from marine natural products as a CDK4/6 inhibitor by means of a structure-based pharmacophore model, molecular docking, ADMET analysis and kinetic simulation; Preliminary analysis indicated that compound **50843** is a potential small-molecule inhibitor that helps inhibit CDK4/6. Subsequent analysis can further evaluate small molecules through structural modification and experimental techniques to help determine the activity of compounds, thereby providing new drug-like leads for CDK4/6.

## Figures and Tables

**Table 1:** The characteristic composition, the number of true/false positive and negative molecules, and the sensitivity of 10 pharmacophore models were constructed. Feature “H” stands for hydrophobic group, while feature “A”, “D” stand for hydrogen bond acceptor and hydrogen bond donor, respectively.

Pharmacophore	Features	Ranking Score	True Positives	True Negatives	False Positives	False Negatives	Sensitivity
Phar01	HHHDA	55.473	3	8	4	4	0.42857
Phar02	HHHDA	55.280	4	8	4	3	0.57143
Phar03	HHDA	51.607	4	11	1	3	0.57143
Phar04	HHHD	50.761	4	10	2	3	0.57143
Phar05	HHDA	50.129	5	10	2	2	0.71429
Phar06	HHHD	49.714	4	10	2	3	0.57143
Phar07	HHDA	48.862	3	8	4	4	0.42857
Phar08	HHDA	48.828	2	9	3	5	0.28571
Phar09	HHDA	48.726	6	10	2	1	0.85714
Phar10	HHDA	48.442	6	9	3	1	0.85714

**Table 2:** Twenty molecules‘ water solubility, intestinal absorption, hepatotoxicity and CYP2D6 enzyme inhibition descriptor properties.

Name	Solubility	Absorption Level	Hepatotoxic	CYP2D6 Inhibit
Molecule17227	3	3	−9.93282	−10.6775
Molecule35962	3	2	−10.2467	−9.79215
Molecule35945	3	2	−10.3996	−9.79215
Molecule50853	3	3	−9.27768	−11.7400
Molecule5999	3	3	−12.0390	−11.4830
Molecule20551	4	2	−7.04826	−5.29830
Molecule7211	3	3	−11.8022	−10.2513
Molecule5996	3	3	−12.0390	−11.4830
Molecule23671	3	3	−4.67926	−11.4629
Molecule9567	3	3	−27.0804	−9.75238
Molecule**41369**	3	1	−13.8276	−0.02226
Molecule6045	3	3	−19.2113	−10.6996
Molecule33567	3	1	−9.88709	−4.57087
Molecule**50843**	4	3	−13.7281	−8.90702
Molecule6049	3	3	−19.2113	−10.6996
Molecule36157	3	1	−4.28935	−4.45112
Molecule6028	3	3	−19.2113	−10.6996
Molecule22564	3	2	−7.74774	−7.53197
Molecule18748	3	3	−5.33843	−8.68489
Molecule6243	3	3	−11.4714	−9.68009

**Table 3:** Libdock scores of 20 selected molecules and positive control Abemaciclib with CDK4/6.

Molecules	2D Structure	Libdock Score (CDK4)	Libdock Score (CDK6)	Fit Value
Molecule17227	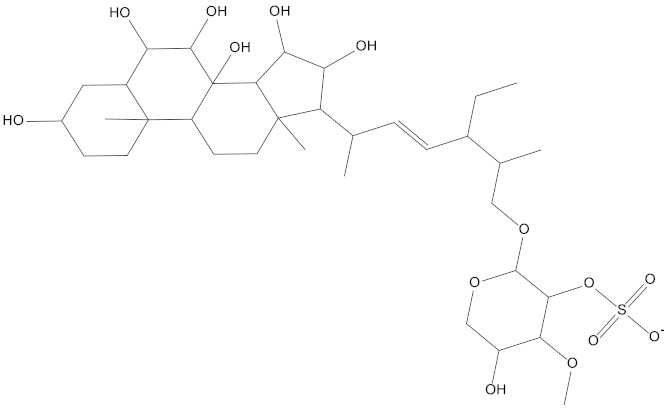	128.538	161.778	3.76592
Molecule35962	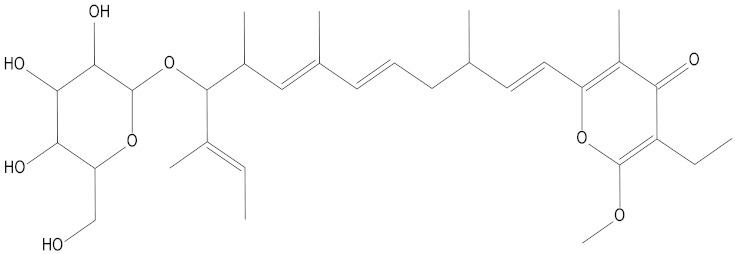	165.215	149.821	3.74528
Molecule35945	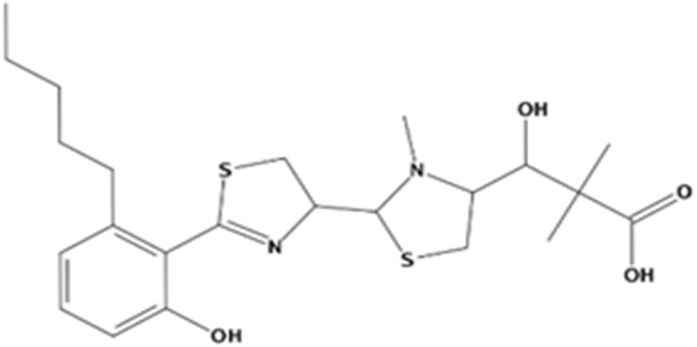	130.659	126.980	3.72383
Molecule50853	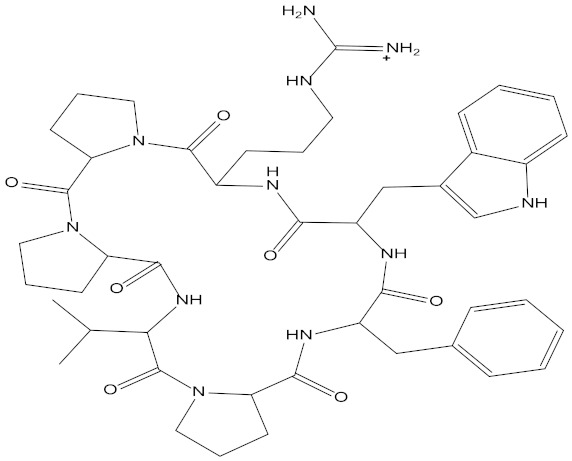	144.891	139.235	3.71061
Molecule5999	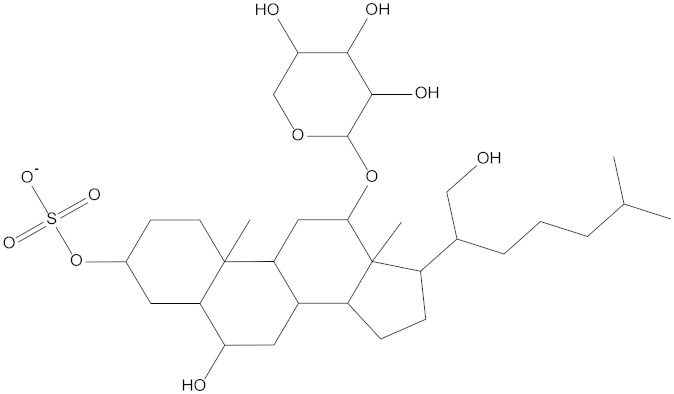	128.538	142.101	3.63037
Molecule20551	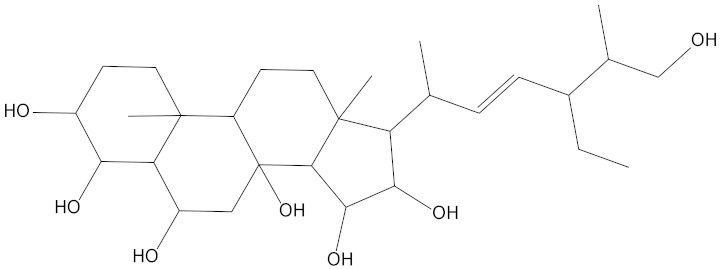	81.8508	124.975	3.62799
Molecule7211	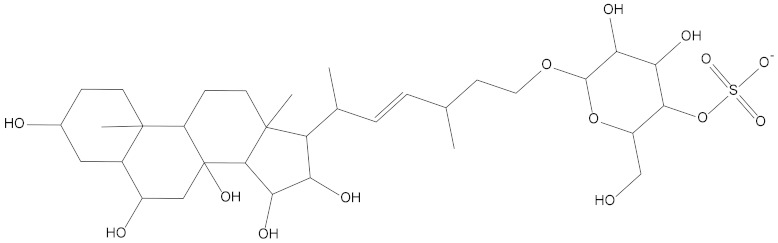	131.753	154.228	3.60959
Molecule5996	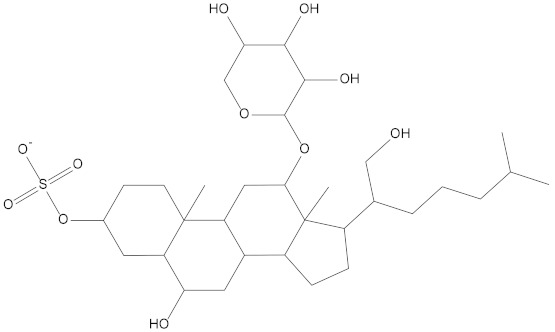	137.638	144.891	3.60816
Molecule23671	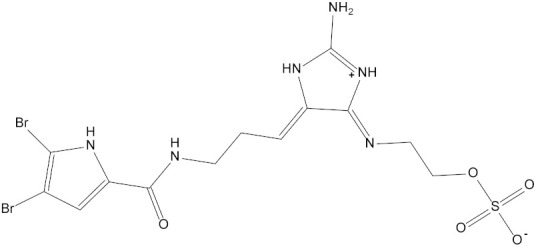	158.406	128.538	3.60593
Molecule9567	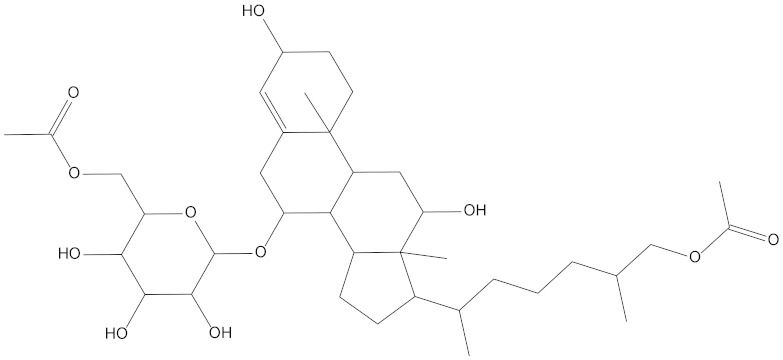	142.101-	165.215	3.58841
Molecule**41369**	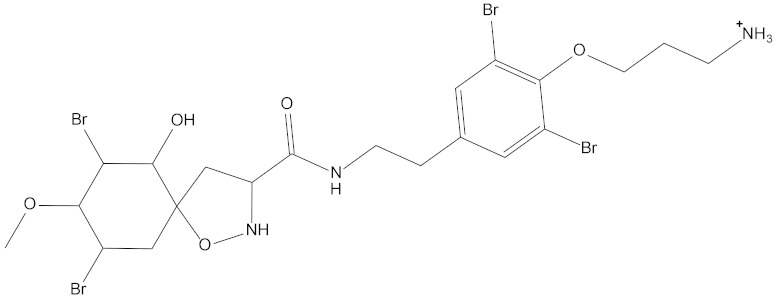	114.793	130.659	3.57953
Molecule6045	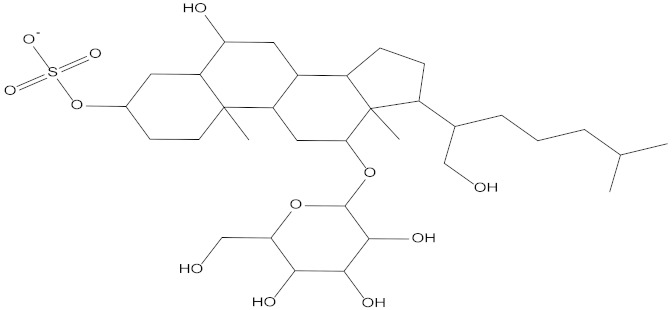	161.778	153.688	3.56692
Molecule33567	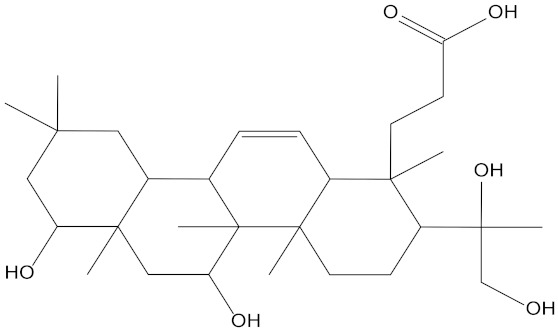	149.821	121.223	3.56079
Molecule**50843**	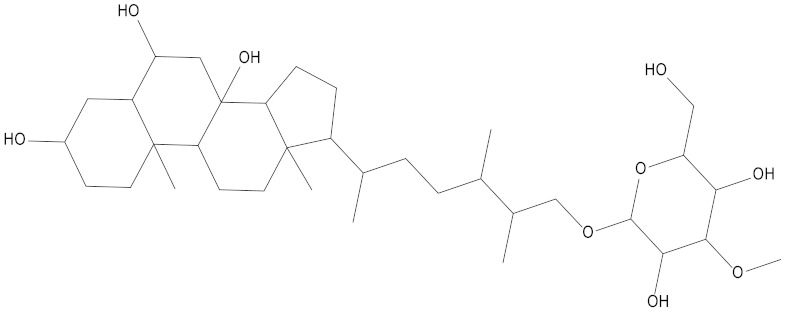	137.62	157.048	3.54233
Molecule6049	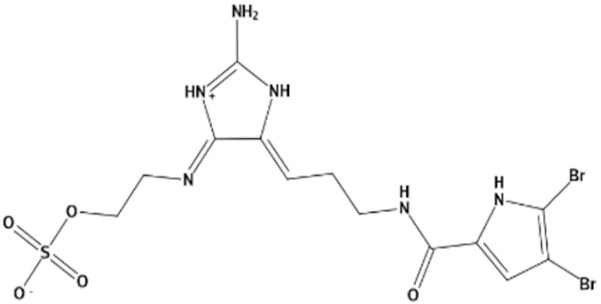	158.406	152.475	3.53563
Molecule36157	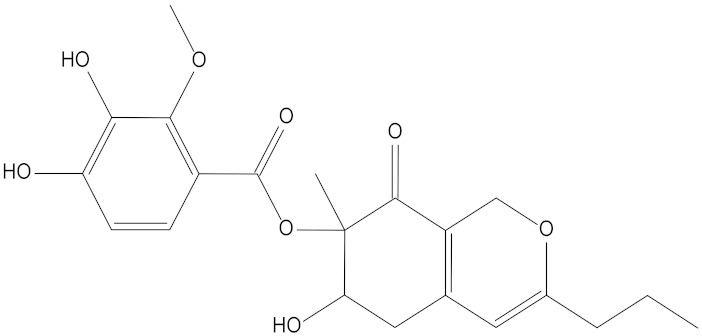	152.076	114.283	3.52724
Molecule6028	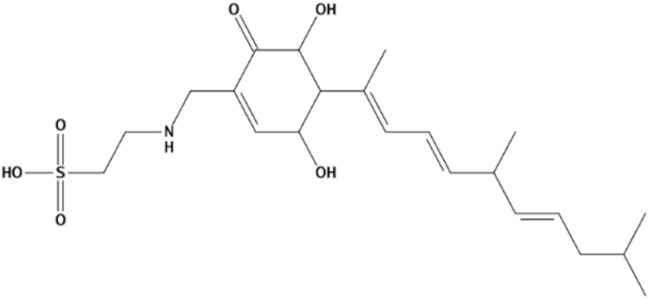	139.235	151.536	3.52680
Molecule22564	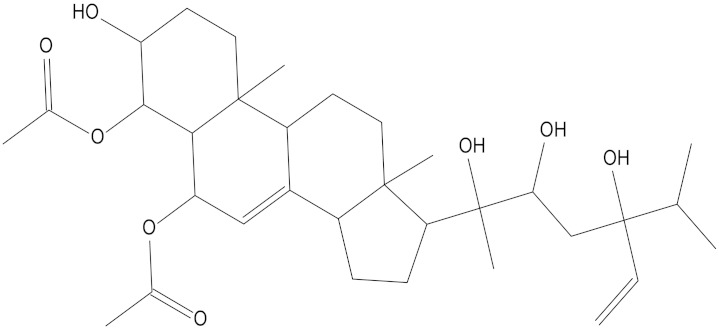	142.101	131.753	3.51793
Molecule18748	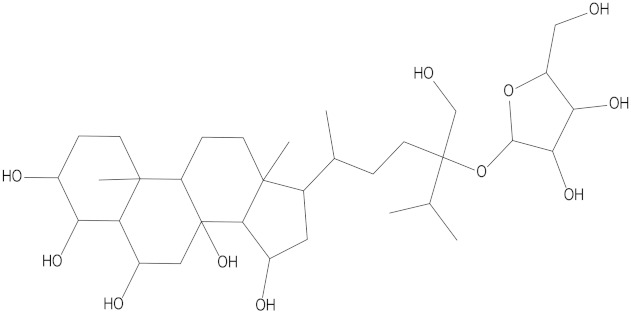	151.536	137.638	3.50622
Molecule6243	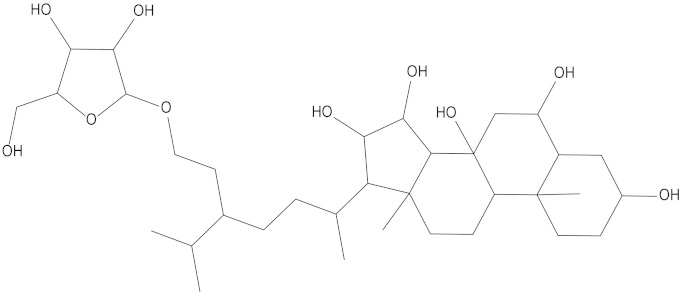	131.753	158.406	3.50202
Abemaciclib	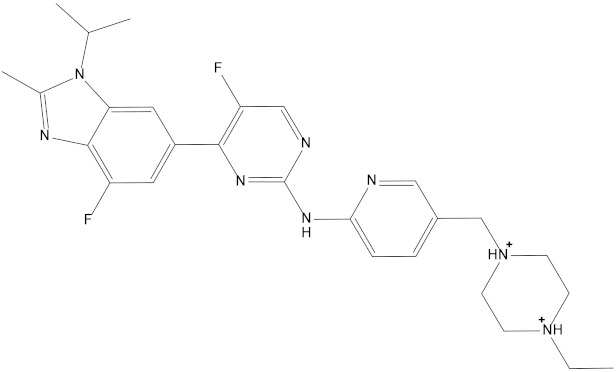	97.7336	152.076	3.46079

**Table 4:** Binding energy of binding for the compound **50843** complexed with CDK4/6.

Pharmacophore	Features	Ranking Score
Van der Waal energy (kJ/mol)	−192.855 ± 90.101	−254.799 ± 51.499
Electrostatic energy (kJ/mol)	−84.560 ± 49.773	−59.732 ± 23.528
Polar solvation energy (kJ/mol)	139.559 ± 111.556	121.507 ± 47.040
SASA energy (kJ/mol)	−16.800 ± 9.461	−19.058 ± 4.394
Binding energy(kJ/mol)	−154.655 ± 39.178	−212.082 ± 42.561

**Table 5:** Synthetic accessibility score parameters (SA score) for molecule **41369** and molecule **50843**.

Molecule	SA Score
**41369**	5.226
**50843**	5.517
Abemaciclib	3.415

**Table 6:** Predicted inhibitory activity of two alternative compounds against tumor cell lines.

Compound	Pa	Pi	Cell Line	Tissue	Tumor Type
**41369**	0.498	0.028	MDA-MB-231	Breast	Adenocarcinoma
**50843**	0.625	0.014	HL-60	Hematopoietic and lymphoid tissue	Leukemia
Abemaciclib	0.750	0.004	LoVo	Colon	Adenocarcinoma

## Data Availability

The data used to support the findings of this study are included within the article.

## Supplementary Materials

The following supporting information can be downloaded at: https://www.mdpi.com/article/10.3390/md20050319/s1, Figure S1: The Crystal structure and Ramachandran plot of CDK4/6.; Figure S2: Five marketed and seven newly published inhibitor structures used to construct/test the pharmacophore; Table S1: The 12 inactive decoy molecule’s smile formula in the test set.
