# Supplementary material for: The Inhibitors of CDK4/6 from a Library of Marine Compound Database: A Pharmacophore, ADMET, Molecular Docking and Molecular Dynamics Study
Source: Mar Drugs. 2022 May 12;20(5):319. doi: 10.3390/md20050319 (PMC9144134; doi:10.3390/md20050319)
Supplement: Supplementary file 1 [file marinedrugs-20-00319-s001.zip › marinedrugs-1662586-supplementary.pdf]

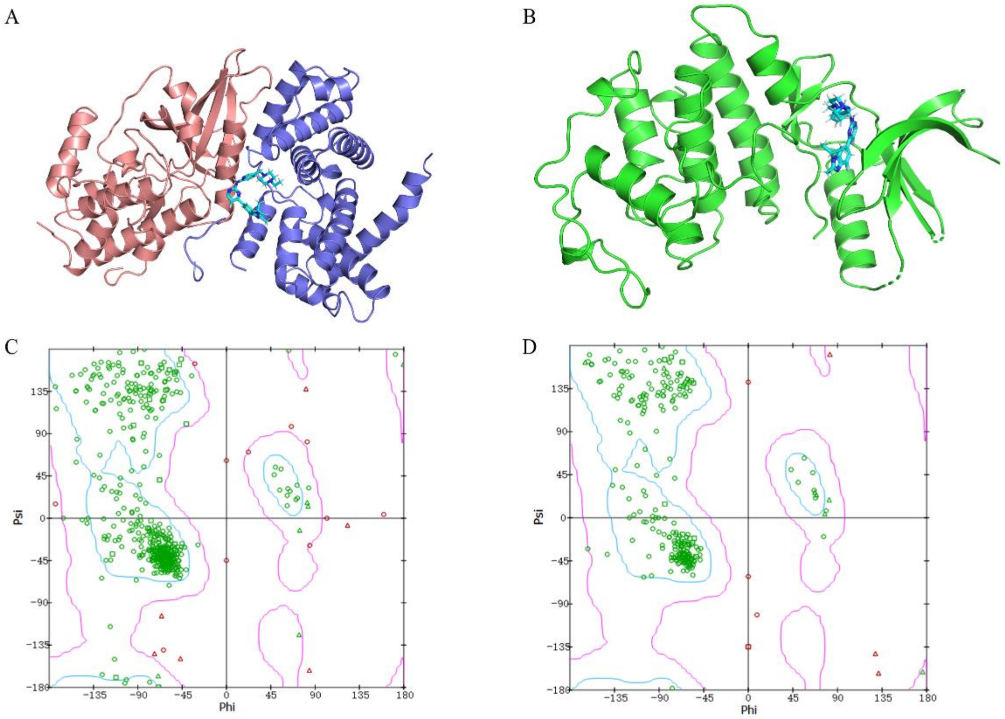

Figure S1. The Crystal structure and Ramachandran plot of CDK4/6. (A) CDK4 (PDB ID:2W96). (B) CKD6（PDB ID:5L2S). (C) The Ramachandran plot of CDK4. (D) The Ramachandran plot of CKD6.

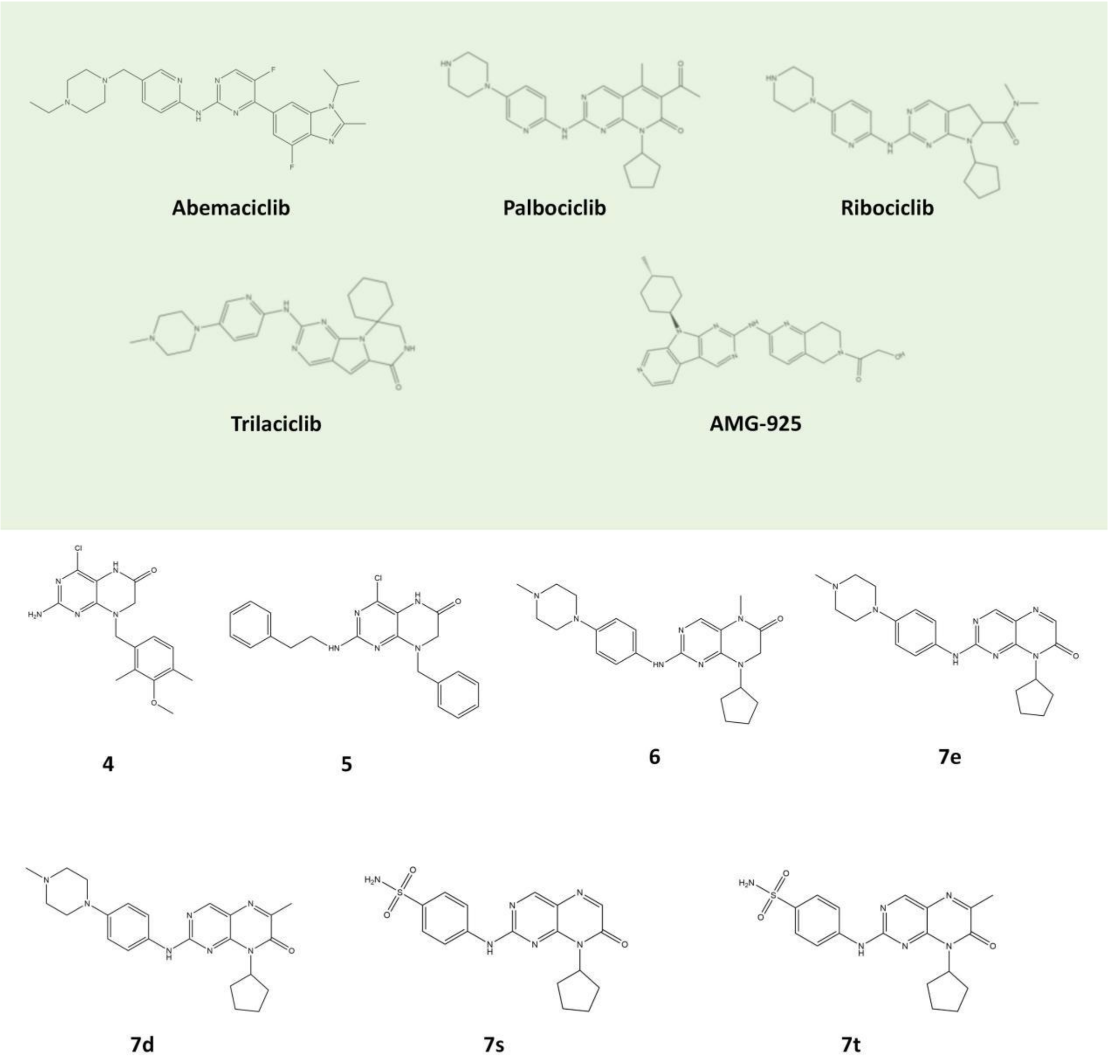

Figure S2. Five marketed and seven newly published inhibitor structures used to construct/test the pharmacophore.

Table S1. The 12 inactive decoy molecule's smile formula in the test set.

	Smile
Decoy molecules	CC1CCC(CC1)n2c3cnccc3c4c2nc(nc4)Nc5ccc6c(n5)CCN(C6)C(=O)CO
	Cc1cc(nn1C)C(=O)N[C@H]2C[C@H]3C(=O)Nc4ccc(cc4C(=O)N3C2)c5cccc(c5)C(F)(F)F
	Cc1ccccc1n2c(=O)c(c([nH]c2=O)O)/C=C\3/C(=NN(C3=O)c4cccc(c4)Cl)C
	Cc1cc2c(cc1NC(=S)NC(=O)c3ccc4c(c3)OCCO4)nn(n2)c5ccc(cc5)F
	Cc1ccc(cc1)S(=O)(=O)Nc2nc(c(s2)C(=O)Nc3ccc4c(c3)OCCO4)C
	COc1cccc(c1)NC(=O)[C@@H]2CCCc3c2nc(s3)NC(=O)c4ccc5c(c4)OCO5
	c1ccc2c(c1)C=C(CO2)C(=O)Nc3ccc(cc3)NS(=O)(=O)c4ccc5c(c4)OCCCO5
	c1cc(cc(c1)NS(=O)(=O)c2cccc(c2)C(=O)Nc3ccc4c(c3)OCCCO4)C#N
	Cc1cc(cc(c1)NC(=O)c2cc(=O)[nH]c3c2cc(cc3)S(=O)(=O)N4CCOCC4)C
	CC(=O)Nc1ccc(cc1)S(=O)(=O)Nc2ccc3c(c2)S(=O)(=O)c4ccccc4O3
	Cc1cc(c2c(c1)sc(n2)NC(=O)c3ccc(=O)n(c3)CC(=O)Nc4nccs4)C
	c1ccc(c(c1)C#N)NC(=O)Nc2cc(ccc2N3CCCCC3)S(=O)(=O)N4CCCC4
